# Benchmarking Survival Outcomes Following Surgical Management of pT3 and pT4 Cutaneous Squamous Cell Carcinoma of the Head and Neck

**DOI:** 10.1245/s10434-022-11669-z

**Published:** 2022-04-13

**Authors:** Amanda E. Yung, Gareth Crouch, Alexander H. R. Varey, Serigne Lo, Michael S. Elliott, Jenny Lee, Robert Rawson, Ruta Gupta, Angela M. Hong, Jonathan R. Clark, Sydney Ch’ng

**Affiliations:** 1 Sydney Medical School, University of Sydney, Sydney, NSW Australia; 2 Royal Prince Alfred Hospital Institute of Academic Surgery, Camperdown, NSW Australia; 3 Faculty of Medicine and Health, The University of Sydney, Sydney, NSW Australia; 4 Melanoma Institute Australia, University of Sydney, Sydney, NSW Australia; 5 Department of Plastic and Reconstructive Surgery, Westmead Hospital, Sydney, NSW Australia; 6 Sydney Head and Neck Cancer Institute, Chris O’Brien Lifehouse Cancer Centre, Sydney, NSW Australia; 7 Department of Medical Oncology, Chris O’Brien Lifehouse Cancer Centre, Sydney, NSW Australia; 8 NSW Pathology, Sydney, NSW Australia; 9 Department of Radiation Oncology, Chris O’Brien Lifehouse Cancer Centre, Sydney, NSW Australia; 10 Department of Plastic and Reconstructive Surgery, Royal Prince Alfred Hospital, Sydney, NSW Australia

## Abstract

**Background:**

pT3/4 head and neck cutaneous squamous cell carcinomas (HNcSCCs) are associated with poor outcomes, including local recurrence, metastasis and death. Whilst surgery remains the standard treatment for advanced HNcSCC, novel systemic therapies, such as immunotherapy, are being used earlier in the treatment paradigm. It is imperative that the clinical outcomes of surgery are clearly described so that conventional and emerging treatment modalities can be better integrated and sequenced in the management of pT3/4 HNcSCC.

**Methods:**

Patients with confirmed pT3/4 HNcSCC undergoing curative surgical resection between 2014-2020 were identified retrospectively from a prospectively maintained research database. The primary outcomes of interest were locoregional control (LRC), disease-specific survival (DSS), and overall survival (OS). The secondary outcome was surgical complication rate.

**Results:**

A total of 104 patients (median age 74, range 41–94 years) were included, 90% of which had pT3 tumors; 36.5% received adjuvant radiotherapy. Median follow-up was 24.3 (range 1.0–84.3) months. LRC at 5 years was 62.0%, DSS at 5 years was 83.7%, and OS at 5 years was 71.9%. Median time to recurrence was 8.4 months. LRC was reduced in the presence of margin involvement and previous treatment (radiotherapy/surgery). The major surgical complication rate was 9.6%.

**Conclusions:**

More than 60% of patients treated surgically for pT3/4 head and neck cSCC were alive and free of disease at 5 years posttreatment. High-risk features such as margin involvement and having had previous treatment (radiotherapy/surgery) should be used to guide adjuvant therapy.

Head and neck cutaneous squamous cell carcinoma (HNcSCC) is a major concern in countries with predominantly Caucasian populations and high ultraviolet (UV) exposure.^1^ The sun exposed regions of the head and neck are the most common sites for advanced cutaneous squamous cell carcinoma (cSCC). Whilst surgery for early lesions is usually straightforward (in up to 97% of patients), advanced lesions not uncommonly require extensive surgical resection, with or without adjuvant (chemo)radiotherapy, and complex reconstruction.^2–4^ Achieving adequate margins can be challenging due to anatomical constraints with nearby critical structures and in cosmetically sensitive facial subsites. As such, some patients with locally advanced disease or matted nodal metastatic disease may be considered incurable with surgery and/or radiotherapy^5^—in cases where local therapy has no prospect of achieving microscopic clear margins, multiple-recurrent disease, or where functional/aesthetic impairment is unacceptable. The majority of these cases are unsurprisingly pT3 or pT4. Until relatively recently, alternatives to radical surgery for advanced HNcSCC, such as definitive radiotherapy ± chemotherapy (cisplatin and carboplatin), have been much inferior to surgery, and were associated with significant adverse events.^5–8^ In September 2018, the immune checkpoint inhibitor (ICI) cemiplimab was approved in the United States and the European Union for patients with unresectable locally advanced or metastatic cSCC who are not candidates for curative surgery or radiotherapy. Approval was granted based largely on positive results in this patient cohort from the pivotal phase II EMPOWER-CSCC trial.^9^ More recently in July 2021, pembrolizumab was approved for patients with locally advanced cSCC who are not candidates for curative surgery or radiotherapy based on encouraging response rates from the Keynote-639 (NCT03284424) phase II trial.^10^ In addition, cemiplimab was also recently trialed as neoadjuvant therapy in 20 stage III/IV (M0) cSCC patients, where treatment was well-tolerated and resulted in a complete or major pathological response rate in 70% of patients.^11^

Emerging systemic therapies will alter the clinical course of advanced HNcSCC once they are integrated into the mainstream treatment. Consequently, it is essential that before this occurs, a benchmark is established for the clinical outcomes of the current standard of care, i.e., surgery and postoperative adjuvant radiotherapy (PORT). Accordingly, this study was designed to describe the real-world outcomes, including complications and prognosticators of recurrence and survival, in a large dataset of patients with pT3/4 HNcSCC in the current era. This will provide an important baseline for comparison in future studies.

## Methods

Patients with histopathology-proven HNcSCC treated between February 2014 and December 2020 were identified from a prospectively maintained research database. Cases before 2018 were restaged by using the AJCC 8th edition staging system. Patients were included if the primary tumor was categorized as pT3 or pT4 and had been treated with curative intent using surgery with or without PORT. In patients presenting with *potentially* local recurrence where the primary tumor had been treated elsewhere, their lesion on presentation to our institution was considered as the index lesion for this analysis. Patients whose primary tumor had been treated elsewhere and subsequently presented to our institution with only regional recurrence (i.e., parotid or cervical nodal metastases) were excluded.

Demographic and clinical data collected included age, gender, previous treatment, and immunosuppression (from solid organ transplantation or hematological malignancy, e.g., chronic lymphocytic lymphoma). Data collected on pathological characteristics included lesion site, histologic margins, tumor depth and differentiation, presence of perineural or lymphovascular invasion, and pathologic nodal status. Clinical data collected included date and method of surgical ablation and reconstruction, in-hospital complications and any revision operations (for esthetics or function or both), use of adjuvant or neoadjuvant therapy, disease recurrence and date of last follow-up or death. For the purpose of this study, radical resection was defined as ablation that included lateral temporal bone resection, orbital exenteration, calvarial resection, maxillectomy, or mandibulectomy. In-hospital complications were classified using the Clavien-Dindo system. If lesions underwent removal with burring of the underlying bone, they were excluded from margin or tumor depth analyses. The data was obtained from a prospectively maintained database supplemented with review of patients’ clinical records and pathology reports where required. All patients had consented for use of their clinical data, with ethics review granted by Sydney Local Health District Ethics Committee, HREC reference number 2019/ETH06423 (X17-0268).

Statistical analyses were performed using SPSS version 26.0 (SPSS, Chicago, IL) and R version 4.0.0 (R Core Team, R Foundation for Statistical Computing, Vienna, Austria, 2020). *P* values <0.05 were considered statistically significant. The primary outcomes were locoregional control (LRC), defined as the absence of any local or regional disease recurrence; disease-specific survival (DSS), defined as absence of death due to HNcSCC; and overall survival (OS), defined as absence of death from any cause. LRC, DSS, and OS were described by using the Kaplan-Meier (KM) method. Log-rank tests were used to estimate the association of independent factors with the primary outcomes. Univariate Cox analysis was used to calculate hazard ratios (HR) if the proportionality hazards assumption holds based on assessment of the Kaplan-Meier curves. Variables with a *p* value ≤ 0.20 on univariate analysis were entered into a multivariate Cox hazard ratio (HR) model to calculate adjusted HR and corresponding 95% confidence intervals (CI). Further Kaplan-Meier analysis and log-rank tests were used to investigate patient subsets who had or had not undergone previous treatment prior to presentation to our institution. The secondary outcome was complication rates. Fisher’s exact test was used to assess for associations between complication rate and nominal variables including performance of radical resection, free flap reconstruction, age ≥65 years, radiotherapy, or medical therapy.

## Results

### Demographic and Tumor Characteristics

A total of 104 patients were included for analysis, including 78 (75%) men. Patient demographics, tumor characteristics, procedures, and complications are shown in Table 1 and Appendix Table 4. The median age at diagnosis was 74 (range 41–94) years. Ninety-four patients (90.4%) presented with pT3 disease, while 10 (9.6%) patients presented with pT4 disease. Eighty-nine patients (85.6%) presented with stage III disease, whereas 15 patients (14.4%) presented with stage IV disease. Thirty-three patients presented with lesions that had previously been treated by external providers. There were 19 neck dissections, of which 9 were therapeutic and the remaining were performed to prepare recipient vessels for free-flap reconstruction. Of the nine patients who underwent therapeutic neck dissection, one patient was found to be N1, one patient was N2a, and seven patients were N3b. The median tumor depth was 7 (range 1–25) mm. Seventeen patients underwent reexcision for involved margins. In four of these patients, microscopic clear margins were not obtained even after reexcision. Of these four patients, two had PORT but still experienced local recurrence, one was eligible for but declined PORT and experienced local recurrence, and one had no PORT with no recurrence.

**Table 1.** Demographic and tumor characteristics in our cohort

	*n* (%) unless otherwise specified
Total	104
*Gender*	
M	78 (75.0)
F	26 (25.0)
*Age (Median, range)*	74 (41-94)
*Immunocompromised*	*18 (17.3)*
*Overall stage*	
III	89 (85.6)
IV	15 (14.4)
*pT stage*	
3^a^	94 (90.4)
4^b^	10 (9.6)
*Previous history of radiotherapy to region*	15 (14.4)
*Adjuvant therapy*	
Radiotherapy	38 (36.5)
Chemotherapy	2 (1.9)
*Tumor characteristics*	
Perineural invasion (PNI)	40 (38.5)
Clinical PNI of facial nerve Clinical PNI of trigeminal nerve	2 (1.9) 5 (4.8)
Lymphovascular invasion	9 (8.7)
Both perineural and lymphovascular invasion	18 (17.3)
Poorly differentiated	34 (32.7)
Moderately differentiated	64 (61.5)
Well differentiated	4 (3.8)
Tumor diameter (median, IQR)	23.5 (23.8)
Tumor depth (median, IQR)	9.0 (7.9)
*Lesions undergoing re-excision*	17 (16.3)
*Final involved margins*	4 (3.8)
*Recurrences*	26 (25.0)
Local	12 (11.5)
Regional	12 (11.5)
Distant	2 (1.9)

^a^pT3 denotes a tumor with greatest tumor dimension ≥4 cm or minimal erosion of the bone or perineural invasion or deep invasion, under the AJCC Cancer Staging Manual, 8th edition

^b^pT4 denotes a tumor with extensive cortical or medullary bone involvement (T4a) or invasion of the base of the cranium or invasion through the foramen of the base of the cranium (T4b) under the AJCC Cancer Staging Manual, 8th edition

### LRC, DSS, and OS

**Fig. 1:**
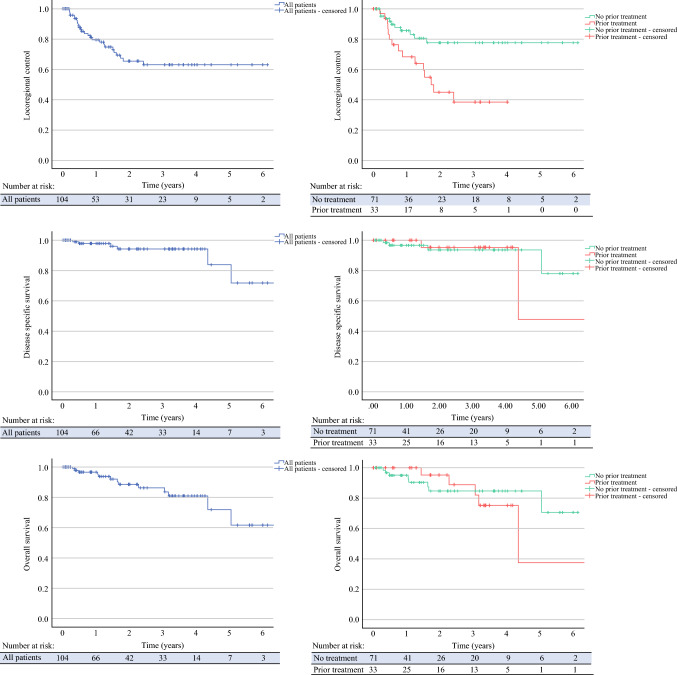
Kaplan-Meier curves demonstrating locoregional control, disease specific survival and overall survival in a cohort of 104 patients with advanced cSCC of the head and neck

Median follow-up was 24.3 (range 1.0–84.3) months. There were 26 recurrences, of which 12 were local, 12 were regional, and 2 were distant. Median time to recurrence was 8.4 (2.4-29.4) months. Of the local recurrences, 11 patients underwent further excision and 1 patient received only palliative chemotherapy. Of the regional recurrences, five patients underwent therapeutic neck dissection and PORT, two had therapeutic neck dissection and postoperative chemotherapy, one had neck dissection only, one had immunotherapy (cemiplimab) only, one had radiotherapy only, and two were palliated. Both patients with distant metastases received palliative medical therapy. Of the 16 patients who underwent lesion removal with burring of underlying bone, 3 developed local recurrence and 1 had regional recurrence in the neck. There were 13 deaths overall, of which 6 were disease-related. The median times to death and disease-specific death were 19.8 months and 6.0 months, respectively. LRC at 5 years was 62.0% (95% CI 49.7-74.3), DSS at 5 years was 83.7% (95% CI 63.7-100.0), and OS at 5 years was 71.9% (95% CI 52.7-91.1) (Fig. 1).

LRC was significantly reduced in patients with an involved primary tumor margin (*p* = 0.002) and prior treatment (radiotherapy/surgery) (*p* = 0.021) (Fig. 1). On multivariate Cox regression, LRC remained significantly reduced in both patients with margin involvement (HR = 4.3, 95% CI 1.2-15.6, *p* = 0.028) and patients with prior treatment (HR = 2.6, 95% CI 1.2-5.7, *p* = 0.019; Table 2). DSS and OS were not significantly associated with any factor (Appendix, Tables 6 and 7).

**Table 2.** Univariate and multivariate analyses of factors affecting locoregional control. Only factors included in the multivariate analysis are shown. For all factors considered in this study, refer to Appendix Table 5

	Univariate analysis			Multivariate analysis		
Factor	Hazard ratio	95% CI	*p* value	Hazard ratio	95% CI	*p* value
Margin involvement	6.049	1.692-21.624	0.006*	2.018	0.744-5.474	0.168
Previous radiotherapy	2.607	1.040-6.533	0.041*	4.363	1.109-17.164	0.035*

Interestingly, patients who presented with synchronous pT3 or pT4 primary lesion and regional (parotid/neck) metastases (*n* = 12) did not have significantly worse LRC, DSS, or OS compared with the rest of the cohort.

### Complications

There were 27 postoperative complications, of which 10 were classified as Clavien-Dindo IIIb, which required a return to the operating room. Postoperative complications of all grades were associated with performance of radical resection (*p* = 0.03) and free-flap reconstruction (*p* = 0.005) but not age ≥65 years, prior treatment, PORT, or adjuvant chemotherapy. Functional complications were experienced by 11 patients, such as ectropion, brow ptosis, and oral incompetence. Ten of these patients had surgery to improve function (Table 3).

**Table 3.** Complications of surgery for cutaneous SCC of the head and neck in our cohort

Complications	*n* (%)
*Surgical complications*	
Clavien-Dindo grade I: wound dehiscence, flap ulceration, facial nerve palsy, partial flap necrosis, flap oedema, seroma	14 (13.5)
Clavien-Dindo grade II: wound infection, superior sagittal sinus thrombosis	3 (2.9)
Clavien-Dindo grade IIIb: postoperative fall and fracture, failed skin graft requiring further free-flap repair, hematoma requiring return to theatre, infected cranioplasty, pedicled flap failure	10 (9.6)
*Functional complications*	
Ectropion requiring revision surgery	5 (4.8)
Brow ptosis requiring brow lift surgery	4 (3.8)
Facial nerve palsy requiring gold weight insertion in eyelid	1 (1.0)
Oral incompetence requiring tendon graft re-suspension	1 (1.0)

## Discussion

This is the largest retrospective data set of 104 advanced HNcSCC patients treated with standard of care of surgery with/without postoperative radiotherapy/medical therapy with long term follow-up before wider use of ICI immunotherapy. In our patient cohort, LRC was reduced by margin involvement and prior treatment. Our patients demonstrated robust survival outcomes with 62.0% LRC, 83.7% DSS, and 71.9% OS at 5 years.

### Demographic Characteristics

Our study population is reflective of the demography typically affected by HNcSCC. The proportion of patients who underwent PORT is relatively low at 36.5%, which was likely, because some patients had already undergone radiotherapy before presentation to our institution (either as primary radiotherapy or in an adjuvant setting following previous surgery by an external provider), and because treatment decision-making has changed with time. For example, at our institution, the previous threshold for PORT to be considered was perineural invasion of a nerve caliber of at least 0.5 mm before the release of AJCC 8^th^ edition, whereas the current threshold is 0.1 mm. While PORT was not shown to affect LRC in our cohort, this was likely due to selection bias, as the benefit of PORT is well established in this regard.^12^

Notably, LRC plateaued after 3 years. However, DSS and OS continued to decrease and had not plateaued at 5 years, most likely explained by the high proportion of elderly patients in our cohort. Interestingly, the main facial subtype involved in our study was the scalp as compared with other studies that reported a predominance for the nose or ear.^7,13^ This probably reflects differential local referral patterns, and explains the relatively high rate of burring in our series, i.e., burring of outer table of the cranium for cases where the deep margin was narrow but where gross/microscopic bone invasion was not present. Immunosuppression for solid organ transplant and hematological malignancy was found to have no effect on survival outcomes. This was an unexpected finding as the association between immunosuppression and worse disease outcomes has been documented in several previous studies including those from our institution, but this was likely due to the low number of patients who were immunosuppressed in our cohort.^7,14,15^ Molecular or genetic markers may offer greater accuracy in identifying aggressiveness of disease compared with morphological characteristics (this is beyond the scope of this article, but is an active area of research at our institution).^16^

### Comparison with Emerging Therapies

Surgery and PORT is current standard of care for advanced cSCC, with concurrent chemoradiotherapy being routine for some institutions in cases where there is extranodal extension in regional metastasis.^5^ With emerging new systemic therapy options for advanced cSCC, benchmarking the current “gold standard” treatment against potential treatment alternatives for advanced HNcSCCs and determining how these alternatives may function as replacement primary or (neo)adjuvant treatment is essential for optimal multidisciplinary discussion and informed consent.

Multiple trials have investigated the role of various medical therapies for advanced cSCC (Appendix Table 8). In particular, the EMPOWER-CSCC trial (NCT02760498) is a landmark study providing strong evidence for the programmed-cell death receptor (PD)-1 inhibitor cemiplimab as monotherapy in cSCC patients, leading to the approval of cemiplimab in the USA for patients with locally advanced or metastatic cSCC who are not candidates for curative surgery or radiotherapy.^17^ Successive reports from this trial have demonstrated durable responses, with a recent update by Rischin et al. reporting an overall response rate of 54.4% across all groups with metastatic or locally advanced cSCC (median follow-up of 15.7 months, 18.5 months or 15.5 months in the 3 groups of the study).^18^ Previously reported data from this trial also demonstrated disease control rates of 62-67.8% across the study cohorts (median follow-up of 9.3 months, 8.1 months or 16.5 months in the 3 groups).^9,19^ Keynote-629, a phase II trial of the adjuvant PD-1 inhibitor pembrolizumab in patients with locally advanced or metastatic cSCC, demonstrated an objective response rate of 34.3%.^10^

Whether ICI immunotherapy will boost survival outcomes when integrated as postoperative adjuvant therapy is currently under investigation. In our cohort, prognostic factors, such as margin involvement and previous treatment (radiotherapy/surgery), did confer poorer survival, so adjuvant immunotherapy may be beneficial. Indeed, Koyfman and colleagues reported a phase II study in patients with recurrent HNcSCC after resection, showing that the PD-1 inhibitor pembrolizumab combined with intensity modulated radiotherapy (IMRT) was safe, with none of the 11 patients experiencing recurrence at time of report.^20^ The Keynote 630 trial (NCT03833167), a phase III trial of adjuvant PD-1 inhibitor pembrolizumab versus placebo in patients with high-risk, locally advanced cSCC following surgery and postoperative radiotherapy, is currently recruiting.^21^ ICI in the neoadjuvant setting also has shown encouraging results. Gross et al. reported on their phase II trial of neoadjuvant cemiplimab in 20 stage III/IV (M0) HNcSCC patients who were planned for surgery and radiation. Neoadjuvant cemiplimab induced a pathologic complete response or major pathology response in 70% of patients, with 11 (55%) patients undergoing treatment de-escalation (omission of PORT), and no disease recurrence was observed in these patients at a median follow-up of 3.8 (range 1.5–11.2) months.^11^

It is important to appreciate that our patient population reflects the real-world situation where patients have poor performance status or are immunocompromised, typically excluded from clinical trials.^22^ Other factors also limit direct comparison of outcomes. Most ICI clinical trials recruit cSCCs across all body subsites,^22^ whilst our study focused on the head and neck, which is an anatomically more challenging region in terms of obtaining a wide excision margin and risk of perineural spread along named nerves. The pattern of disease among the study subjects, including locally advanced disease only, regional metastasis only or distant metastasis, is an important consideration when scrutinizing survival data. Our study has a preponderance of locally advanced disease thereby preventing any comparison with the metastatic cohort. Moreover, the definition for “advanced cSCC” varies between studies.^22^

Of note, there is not a large published series of pT3/4 cSCC patients treated with primary ICI. At present, the real-world outcomes of primary ICI in locally advanced cSCC patients are still emerging. Hanna and colleagues reported that in a cohort of 61 patients with advanced cSCC patients treated with various ICIs, the best overall response was lower at 31.5% compared with trial data at a median follow-up of 8.5 months.^23^ Similarly, another study of 74 patients with advanced cSCC treated with cemiplimab, pembrolizumab or nivolumab showed an objective response rate of 34% (median follow-up not reported).^24^ On the other hand, Wu and colleagues recently reported on a series of 11 patients with advanced cSCC and clinical PNI treated with ICI therapy, in which 9 patients showed radiographic evidence of perineural disease control (median follow-up 13 months).^25^

### Surgical Complications

The overall risk of severe complications requiring return to theatre was low (8.7%). Complications were more common among those who underwent radical resections and who required more complex reconstruction using free flaps. This suggests the risk of complications and associated morbidity is proportional to tumor dimension and operative complexity. Patients eligible for more complex surgery should be informed of the greater risk of complications requiring return to theatre, such as wound complications. In addition, return to theatre may be required for reexcision of lesions with involved margins, which was seen in 16.3% of our cohort. The final proportion of patients in whom clear microscopic margins were unachievable was low at 3.8%. We acknowledge that as a retrospective study, this may not be achievable for all pT3 and pT4 lesions and likely reflects balanced multidisciplinary team discussion and excellent patient selection.

It is worth noting that simple reconstruction with direct closure or skin grafting was possible in 32% of subjects, implying many patients with locally advanced HNcSCC by stage are subject to low surgery risks. Reconstructive surgeons therefore play a crucial role in decision-making surrounding treatment within a multidisciplinary team, e.g., by identifying patients eligible for simple reconstruction despite advanced T classification, and predicting those who may be surgically treated with minimal morbidity.

### Limitations

Although this is the largest reported patient cohort with pT3/4 HNcSCCs, we appreciate that ours is a selective patient cohort. A small minority of lesions included in our study population, while considered index lesions, were likely recurrent lesions that had previously been treated by an external provider. However, we still considered these index lesions as the differentiation of a recurrent lesion versus a second primary is at times arbitrary, and these lesions were potentially of a lesser T category before referral to our institution. Being retrospective in nature, quantification of presurgical functional status is lacking. Whilst the surgical complications and revisional surgeries reported provide one aspect of morbidity, it does not adequately capture other more subtle morbidities including symptoms experienced in the immediate postoperative phase, such as nausea, fatigue, or psychological morbidity. It also does not capture factors affecting decision making in the treatment selection of many of these patients, including patient’s preference, frailty, and comorbidities.

## Conclusions

Our study showed 62.0% LRC, 83.7% DSS, and 71.9% OS at 5 years, with the current standard of care of surgery ± PORT for pT3/4 HNcSCC. Adjuvant treatment modalities should however be considered/introduced in the sub-group of patients with poor prognostic factors, including margin involvement and prior therapy. Further studies assessing interactions between preoperative functional status and surgical outcomes/complications, and prediction of individual response to ICI immunotherapy, are required to allow better selection of patients most suitable for surgery versus alternative primary or (neo)adjuvant therapies for advanced HNcSCC.

## Appendix

See Tables 4, 5, 6, 7 and 8.

**Table 4.** Further tumor characteristic and procedures undergone

	***n*** (%) unless otherwise specified
*Facial subsite*	
Scalp	27 (26.0)
Forehead inc. temple	17 (16.3)
Ear	11 (10.6)
Nose	9 (8.7)
Cheek	22 (21.2)
Lip	14 (13.4)
Other	4 (3.8)
*Side*	
Right	36 (34.6)
Left	43 (41.3)
Midline	25 (24.0)
*Locoregional disease on presentation*	
Parotid	3 (2.9)
Neck	9 (8.7)
*Resection characteristics*	
Neck dissection (both therapeutic and elective)	19 (18.3)
Parotidectomy (both therapeutic and elective)	8 (7.7)
Craniectomy	5 (4.8)
Mandibulectomy	3 (2.9)
Maxillectomy	2 (1.9)
Burring of underlying bone	15 (14.4)
Temporal bone resection	4 (3.8)
Orbital exenteration	2 (1.9)
Full thickness eyelid resection	3 (2.9)
Rhinectomy	6 (5.8)
Full-thickness lip resection	16 (15.4)
Full-thickness ear resection	10 (9.6)
Cheek excision	16 (15.4)
Forehead excision	15 (14.4)
Scalp excision	26 (25.0)
*Reconstructive characteristics*	
Combined free and locoregional flap	3 (2.9)
Locoregional flap	34 (32.7)
Free flap	35 (33.7)
Skin graft only	15 (14.4)
Primary closure only	17 (16.3)

**Table 5.** 1-, 3-, and 5-year probabilities and standard errors for locoregional control (LRC) for various factors, and log-rank *p*-values

	LRC at 1 year (SE)	LRC at 3 years (SE)	LRC at 5 years (SE)	Hazard ratio	95% CI	*p* (log-rank)
	Locoregional Disease on Presentation?					
Yes	0.540 (0.154)	0.540 (0.154)	0.540 (0.154)			0.109
No	0.848 (0.042)	0.642 (0.067)	0.642 (0.067)			
	Stage					
III	0.829 (0.045)	0.652 (0.067)	0.652 (0.067)	2.014	0.806-5.029	0.126
IV	0.593 (0.144)	0.494 (0.150)	0.494 (0.150)			
	Margin Involvement					
Yes	0.533 (0.248)	0.533 (0.248)	0.533 (0.248)	6.049	1.692-21.624	0.002*
No	0.823 (0.043)	0.653 (0.062)	0.653 (0.062)			
	Perineural spread					
Yes	0.738 (0.066)	0.590 (0.087)	0.590 (0.087)	1.414	0.641-3.122	0.388
No	0.864 (0.057)	0.671 (0.088)	0.671 (0.088)			
	Lymphovascular invasion					
Yes	0.620 (0.107)	0.620 (0.107)	0.620 (0.107)			0.330
No	0.852 (0.046)	0.624 (0.075)	0.624 (0.075)			
	Differentiation					
Well	1.00	0.667 (0.272)	0.667 (0.272)			0.682
Moderately	0.805 (0.056)	0.674 (0.071)	0.674 (0.071)			
Poorly	0.718 (0.095)	0.547 (0.128)	0.547 (0.128)			
	Previous radiotherapy					
Yes	0.587 (0.142)	0.294 (0.219)	0.294 (0.219)	2.607	1.040-6.533	0.034*
No	0.831 (0.045)	0.672 (0.063)	0.672 (0.063)			
	Adjuvant radiotherapy					
Yes	0.805 (0.066)	0.561 (0.105)	0.561 (0.105)			0.565
No	0.787 (0.0610	0.671 (0.075)	0.671 (0.075)			
	Immunocompromised					
Yes	0.813 (0.098)	0.492 (0.161)	0.492 (0.161)			0.367
No	0.775 (0.062)	0.712 (0.071)	0.712 (0.071)			
	Radical Resection					
Yes	0.748 (0.110)	0.523 (0.156)	0.523 (0.156)			0.420
No	0.803 (0.049)	0.647 (0.067)	0.647 (0.067)			
	N3b					
Yes	0.667 (0.192)	0.667 (0.192)	0.667 (0.192)			0.623
No	0.806 (0.045)	0.631 (0.064)	0.631 (0.064)			
	Facial Subsite					
Scalp	0.716 (0.099)	0.525 (0.120)	0.525 (0.120)	1.544	0.688-3.466	0.288
Forehead	0.821 (0.177)	0.513 (0.190)	0.513 (0.190)	1.153	0.397-3.346	0.794
Ear	0.762 (0.148)	0.610 (0.181)	0.610 (0.181)	0.973	0.293-3.229	0.902
Nose	0.889 (0.105)	0.711 (0.180)	0.711 (0.180)	0.769	0.182-3.249	0.845
Cheek	0.822 (0.093)	0.740 (0.155)	0.740 (0.155)	0.730	0.251-2.119	0.561
Lip	0.779 (0.113)	0.682 (0.134)	0.682 (0.134)	0.811	0.281-2.341	0.858
	Prior treatment (Radiotherapy/surgery)					
Yes	0.682 (0.088)	0.384 (0.107)	0.384 (0.107)	2.886	1.325-6.289	0.005*
No	0.855 (0.048)	0.776 (0.062)	0.776 (0.062)			
	Tumor depth					
≥7 mm	0.803 (0.067)	0.613 (0.098)	0.613 (0.098)	1.351	0.522-3.491	0.533
<7 mm	0.825 (0.080)	0.655 (0.111)	0.655 (0.111)			
	Tumor diameter					
≥20 mm	0.763 (0.074)	0.620 (0.097)	0.620 (0.097)	1.201	0.520-2.775	0.668
<20 mm	0.807 (0.067)	0.640 (0.093)	0.640 (0.093)			

Hazard ratios are displayed only where the proportionality hazard assumption held and univariate Cox analysis was performed

**Table 6.** 1-, 3-, and 5-year probabilities for disease-specific survival (DSS) for various factors and log-rank *p*-values

	DSS at 1 year (SE)	DSS at 3 years (SE)	DSS at 5 years (SE)	Hazard ratio	95% CI	*p* (log-rank)
	Locoregional disease on presentation					
Yes	0.909 (0.087)	0.909 (0.087)	0.909 (0.087)			0.784
No	0.987 (0.013)	0.946 (0.031)	0.827 (0.114)			
	Stage					
III	0.987 (0.013)	0.944 (0.032)	0.809 (0.128)			0.897
IV	0.923 (0.074)	0.923 (0.074)	0.923 (0.074)			
	Margin Involvement					
Yes	1.00	1.00	1.00			0.473
No	0.976 (0.017)	0.938 (0.031)	0.821 (0.113)			
	Perineural invasion					
Yes	0.961 (0.027)	0.961 (0.027)	0.961 (0.027)			0.400
No	1.00	0.920 (0.054)	0.767 (0.147)			
	Lymphovenous Invasion					
Yes	0.911 (0.060)	0.911 (0.060)	0.911 (0.060)			0.133
No	1.00	0.949 (0.035)	0.813 (0.129)			
	Differentiation					
Well	1.00	1.00	1.00			0.889
Moderately	1.00	0.945 (0.038)	0.788 (0.147)			
Poorly	0.932 (0.046)	0.932 (0.046)	0.932 (0.046)			
	Previous radiotherapy					
Yes	1.00	0.875 (0.117)	0.875 (0.117)			0.514
No	0.974 (0.018)	0.952 (0.028)	0.846 (0.103)			
	Adjuvant radiotherapy					
Yes	0.974 (0.026)	0.933 (0.047)	0.622 (0.256)	1.650	0.329-8.261	0.538
No	0.980 (0.020)	0.949 (0.036)	0.949 (0.036)			
	Immunocompromised					
Yes	1.00	0.857 (0.132)	0.857 (0.132)			0.666
No	0.963 (0.026)	0.933 (0.039)	0.622 (0.255)			
	Radical Resection					
Yes	0.938 (0.061)	0.938 (0.061)	0.938 (0.061)			0.672
No	0.986 (0.014)	0.943 (0.033)	0.825 (0.114)			
	N3b					
Yes	0.833 (0.152)	0.833 (0.152)	0.833 (0.152)			0.474
No	0.988 (0.012)	0.950 (0.029)	0.831 (0.114)			
	Facial Subsite					
Scalp	1.00	0.933 (0.064)	0.933 (0.064)			0.382
Forehead	0.923 (0.074)	0.923 (0.074)	0.923 (0.074)			0.852
Ear	0.889 (0.105)	0.741 (0.161)	0.741 (0.161)			0.086
Nose	1.00	1.00	1.00			0.522
Cheek	1.00	1.00	1.00			0.179
Lip	1.00	1.00	1.00			0.280
	Prior treatment (Radiotherapy/surgery)					
Yes	1.00	0.952 (0.046)	0.476 (0.338)			0.861
No	0.966 (0.024)	0.936 (0.038)	0.936 (0.038)			
	Tumor depth					
≥7 mm	0.971 (0.028)	0.920 (0.056)	0.736 (0.171)			0.521
<7 mm	1.00	1.00	1.00			
	Tumor diameter					
≥20 mm	0.971 (0.028)	0.917 (0.059)	0.688 (0.203)	4.548	0.462-44.805	0.157
<20 mm	1.00	1.00	1.00			

Hazard ratios are displayed only where the proportionality hazard assumption held and univariate Cox analysis was performed

**Table 7.** 1-, 3-, and 5-year probabilities for overall survival (OS) for various factors, and log-rank *p*-values

	OS at 1 year (SE)	OS at 3 years (SE)	OS at 5 years (SE)	Hazard Ratio	95% CI	P (log-rank)
	Locoregional Disease on Presentation?					
Yes	0.909 (0.087)	0.808 (0.122)	0.808 (0.122)			0.891
No	0.975 (0.018)	0.868 (0.048)	0.702 (0.109)			
	Stage					
III	0.974 (0.018)	0.878 (0.044)	0.695 (0.119)			0.826
IV	0.923 (0.074)	0.791 (0.138)	0.791 (0.138)			
	Margin Involvement					
Yes	1.00	1.00	1.00			0.337
No	0.965 (0.020)	0.854 (0.047)	0.699 (0.106)			
	Perineural Spread					
Yes	0.961 (0.027)	0.883 (0.061)	0.815 (0.086)			0.379
No	0.974 (0.026)	0.827 (0.071)	0.653 (0.136)			
	Lymphovascular Invasion					
Yes	0.911 (0.060)	0.835 (0.091)	0.835 (0.091)			0.671
No	0.985 (0.015)	0.872 (0.050)	0.685 (0.120)			
	Differentiation					
Well	1.00	0.667 (0.272)	0.667 (0.272)			0.838
Moderately	0.982 (0.018)	0.860 (0.054)	0.684 (0.136)			
Poorly	0.932 (0.046)	0.932 (0.046)	0.799 (0.130)			
	Previous Radiotherapy					
Yes	1.00	0.875 (0.117)	0.438 (0.315)			0.512
No	0.961 (0.022)	0.861 (0.047)	0.741 (0.099)			
	Adjuvant Radiotherapy					
Yes	0.974 (0.026)	0.827 (0.082)	0.501 (0.216)			0.583
No	0.961 (0.027)	0.882 (0.050)	0.840 (0.063)			
	Immunocompromised					
Yes	1.00	0.857 (0.132)	0.857 (0.132)			0.246
No	0.946 (0.031)	0.796 (0.068)	0.497 (0.210)			
	Radical Resection					
Yes	00.838 (0.061)	0.804 (0.134)	0.670 (0.166)			0.385
No	0.973 (0.019)	0.875 (0.045)	0.736 (0.109)			
	N3b					
Yes	0.833 (0.152)	0.833 (0.152)	0.833 (0.152)			0.977
No	0.976 (0.017)	0.684 (0.047)	0.704 (0.107)			
	Facial Subsite					
Scalp	1.00	0.933 (0.064)	0.933 (0.064)			0.449
Forehead	0.846 (0.100)	0.846 (0.100)	0.667 (0.171)			0.376
Ear	0.889 (0.105)	0.494 (0.228)	0.494 (0.228)			0.105
Nose	1.00	1.00	1.00			0.957
Cheek	1.00	0.857 (0.094)	0.714 (0.152)			0.811
Lip	1.00	0.909 (0.087)	0.909 (0.087)			0.356
	Prior treatment (Radiotherapy/surgery)					
Yes	1.00	0.889 (0.075)	0.376 (0.271)			0.756
No	0.949 (0.028)	0.847 (0.055)	0.847 (0.055)			
	Tumor depth					
≥7 mm	0.944 (0.038)	0.811 (0.079)	0.603 (0.154)			0.198
<7 mm	1.00	1.00	0.900 (0.095)			
	Tumor diameter					
≥20 mm	0.971 (0.028)	0.881 (0.067)	0.661 (0.197)			0.873
<20 mm	0.975 (0.025)	0.938 (0.044)	0.824 (0.085)			

Hazard ratios are displayed only where the proportionality hazard assumption held and univariate Cox analysis was performed

**Table 8.** Efficacy outcomes in studies of nonsurgical management of advanced cSCC

Study	Regimen	No. patients	Median follow-up, mo	Response rate (%)	Median OS, mo	Other outcomes
Migden et al. (2018) – phase I study expansion cohorts, EMPOWER-CSCC^22^	Cemiplimab	26	11.0	50 (partial response; PR)	Not reported	Disease control rate (DCR): 65%
Rischin et al. (2020) – phase II study, groups 1 and 3, EMPOWER-CSCC^19^	Cemiplimab	115	8.1 for Group 3, 16.5 for Group 1	33.9 (PR) 11.3 (complete response; CR	Not reached; 80.7% at 12 months	DCR: 67.8%
Migden et al. (2020) – phase II study, group 2, EMPOWER-CSCC^9^	Cemiplimab	78	9.3	13 (CR) 24 (PR)	Not reported	DCR: 62%
Grob et al. (2020) – phase II study, KEYNOTE-629^10^	Pembrolizumab	105	11.4	3.8 (CR) 30.5 (PR)	Not reached; 60.3% at 12 months	DCR: 52.4% Median progression-free survival (PFS): 6.9 months
William et al. (2017) – phase II study, no trial name^26^	Gefitinib	40	Not reported	16 (overall response; OR)	12.9	Median PFS: 3.8 months
Maubec et al. (2011) – phase II study, no trial name^27^	Cetuximab	36	Not reported	28 (OR)	8.1	DCR: 69% Median PFS: 4.1 months
Joseph et al. (2019) – pilot study^28^	Cetuximab + radiotherapy	8	25	25 (PR) 75 (CR)	Not reached; 87.5% at 2 years	
Gold et al. (2018) – phase II study, no trial name^29^	Erlotinib	39	Not reported	10 (PR)	13	DCR: 72% Median PFS: 4.7 months
Hourbeigt et al. (2020) – retrospective study^30^	Panitumumab +/- radiotherapy	25	Not reported	16 (CR) 36 (PR)	10.5	DCR: 32% at 6 months Median PFS: 6.9 months
Shin et al. (2002) – phase II study, no trial name^31^	IFNa, retinoic acid, cisplatin	39	37.9	17 (CR) 17 (PR)	14.6	
Jarkowski et al. (2016) – no trial name^32^	Various systemic therapies: platinum-based, taxane, cetuximab	25	42.8	44 (PR)	10.9	Median PFS: 5.5 months
Hillen et al. (2018) – no trial name^33^	Various systemic therapies: cetuximab, panitumumab, erlotinib	32	Not reported	27 (partial response) 7 (complete response)	Not reported	
Hanna et al. (2020) – no trial name^23^	Various Immune checkpoint inhibitors	61	8.5	31.5 (complete and partial responses)	8	Median PFS: 7 months
